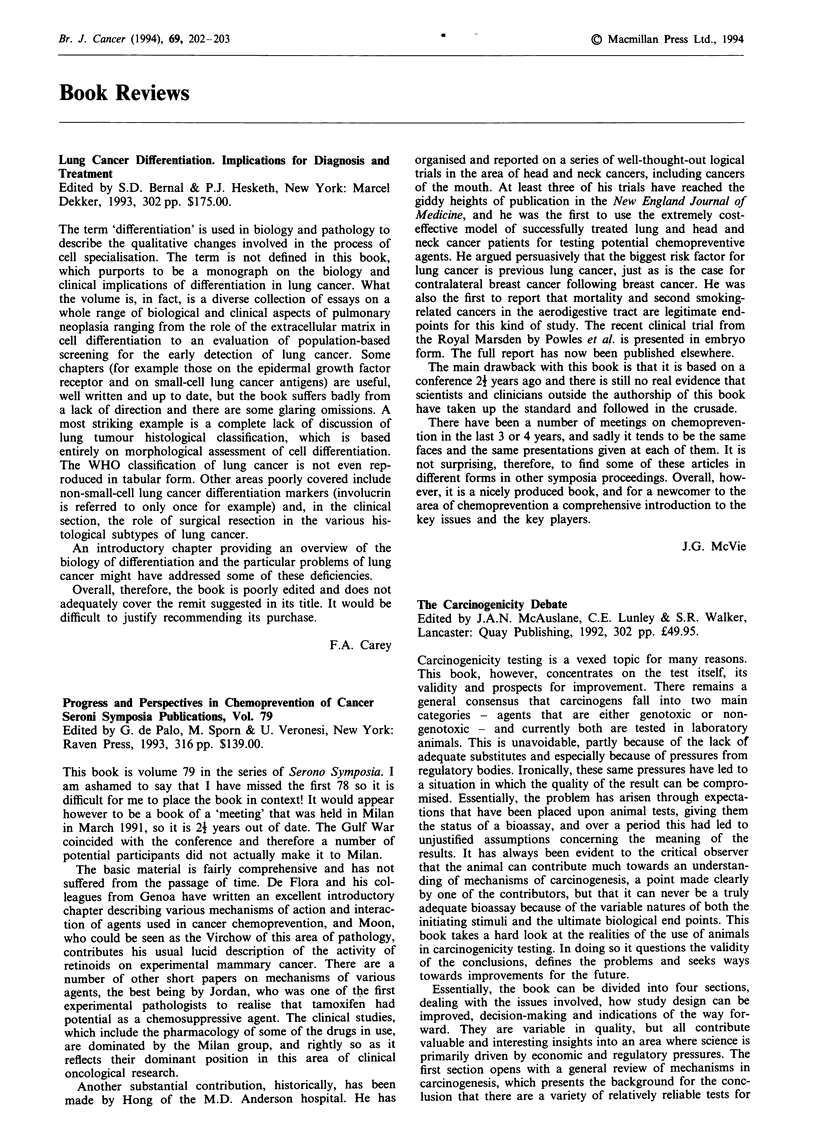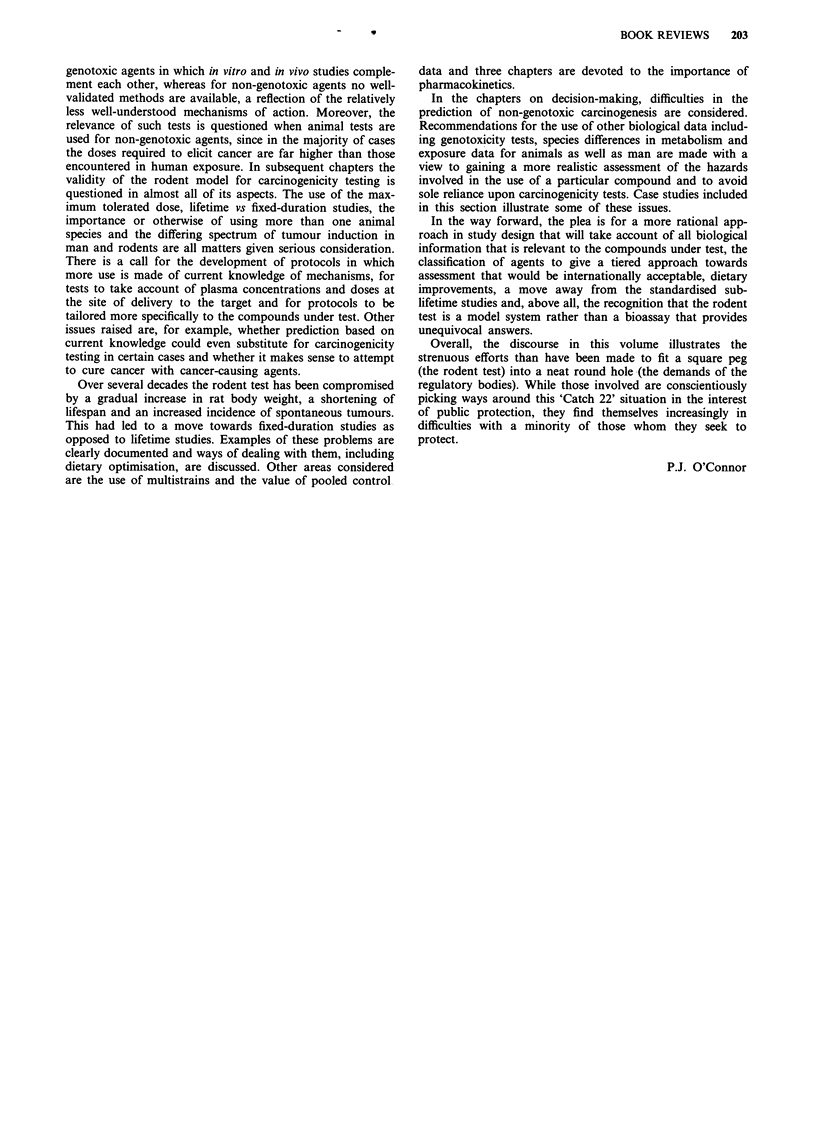# The carcinogenicity debate

**Published:** 1994-01

**Authors:** P.J. O'Connor


**The Carcinogenicity Debate**
Edited by J.A.N. McAuslane, C.E. Lunley & S.R. Walker, Lancaster: Quay Publishing, 1992, 302 pp. £49.95.

Carcinogenicity testing is a vexed topic for many reasons. This book, however, concentrates on the test itself, its validity and prospects for improvement. There remains a general consensus that carcinogens fall into two main categories – agents that are either genotoxic or non-genotoxic – and currently both are tested in laboratory animals. This is unavoidable, partly because of the lack of adequate substitutes and especially because of pressures from regulatory bodies. Ironically, these same pressures have led to a situation in which the quality of the result can be compromised. Essentially, the problem has arisen through expectations that have been placed upon animal tests, giving them the status of a bioassay, and over a period this had led to unjustified assumptions concerning the meaning of the results. It has always been evident to the critical observer that the animal can contribute much towards an understanding of mechanisms of carcinogenesis, a point made clearly by one of the contributors, but that it can never be a truly adequate bioassay because of the variable natures of both the initiating stimuli and the ultimate biological end points. This book takes a hard look at the realities of the use of animals in carcinogenicity testing. In doing so it questions the validity of the conclusions, defines the problems and seeks ways towards improvements for the future.

Essentially, the book can be divided into four sections, dealing with the issues involved, how study design can be improved, decision-making and indications of the way forward. They are variable in quality, but all contribute valuable and interesting insights into an area where science is primarily driven by economic and regulatory pressures. The first section opens with a general review of mechanisms in carcinogenesis, which presents the background for the conclusion that there are a variety of relatively reliable tests for genotoxic agents in which *in vitro* and *in vivo* studies complement each other, whereas for non-genotoxic agents no well-validated methods are available, a reflection of the relatively less well-understood mechanisms of action. Moreover, the relevance of such tests is questioned when animal tests are used for non-genotoxic agents, since in the majority of cases the doses required to elicit cancer are far higher than those encountered in human exposure. In subsequent chapters the validity of the rodent model for carcinogenicity testing is questioned in almost all of its aspects. The use of the maximum tolerated dose, lifetime *vs* fixed-duration studies, the importance or otherwise of using more than one animal species and the differing spectrum of tumour induction in man and rodents are all matters given serious consideration. There is a call for the development of protocols in which more use is made of current knowledge of mechanisms, for tests to take account of plasma concentrations and doses at the site of delivery to the target and for protocols to be tailored more specifically to the compounds under test. Other issues raised are, for example, whether prediction based on current knowledge could even substitute for carcinogenicity testing in certain cases and whether it makes sense to attempt to cure cancer with cancer-causing agents.

Over several decades the rodent test has been compromised by a gradual increase in rat body weight, a shortening of lifespan and an increased incidence of spontaneous tumours. This had led to a move towards fixed-duration studies as opposed to lifetime studies. Examples of these problems are clearly documented and ways of dealing with them, including dietary optimisation, are discussed. Other areas considered are the use of multistrains and the value of pooled control data and three chapters are devoted to the importance of pharmacokinetics.

In the chapters on decision-making, difficulties in the prediction of non-genotoxic carcinogenesis are considered. Recommendations for the use of other biological data including genotoxicity tests, species differences in metabolism and exposure data for animals as well as man are made with a view to gaining a more realistic assessment of the hazards involved in the use of a particular compound and to avoid sole reliance upon carcinogenicity tests. Case studies included in this section illustrate some of these issues.

In the way forward, the plea is for a more rational approach in study design that will take account of all biological information that is relevant to the compounds under test, the classification of agents to give a tiered approach towards assessment that would be internationally acceptable, dietary improvements, a move away from the standardised sub-lifetime studies and, above all, the recognition that the rodent test is a model system rather than a bioassay that provides unequivocal answers.

Overall, the discourse in this volume illustrates the strenuous efforts than have been made to fit a square peg (the rodent test) into a neat round hole (the demands of the regulatory bodies). While those involved are conscientiously picking ways around this 'Catch 22' situation in the interest of public protection, they find themselves increasingly in difficulties with a minority of those whom they seek to protect.

P.J. O'Connor